# Supplementary material for: Lipid vesicles chaperone an encapsulated RNA aptamer
Source: Nat Commun. 2018 Jun 13;9:2313. doi: 10.1038/s41467-018-04783-8 (PMC5998061; doi:10.1038/s41467-018-04783-8)
Supplement: Supplementary file 1 — Supplementary Information [file 41467_2018_4783_MOESM1_ESM.pdf]

Supplementary Information

Lipid Vesicles Chaperone an Encapsulated RNA Aptamer

Saha *et al.*

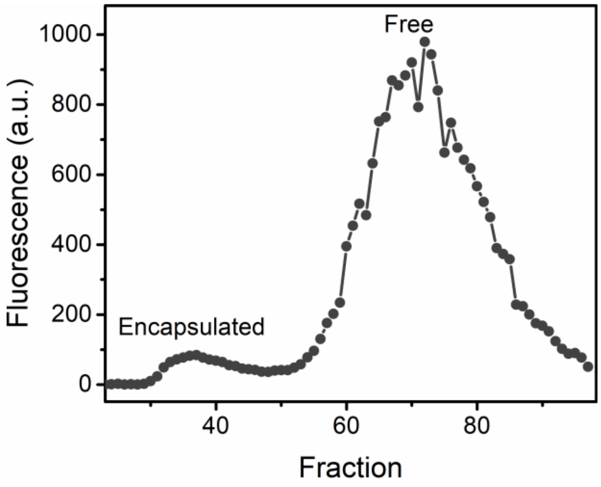

Supplementary Figure 1. Column purification of MA vesicles from unencapsulated RNA. Shown here is the chromatogram of a representative purification of MA vesicles encapsulating MG aptamer bound to MG, using a size exclusion column (Sepharose 4B). The unencapsulated MG-RNA (12 kD; 'Free') is retained by Sepharose 4B and elutes at high fraction number (centered around 70), while MG-RNA encapsulated in vesicles elutes with the void volume at low fraction number (centered around 36).

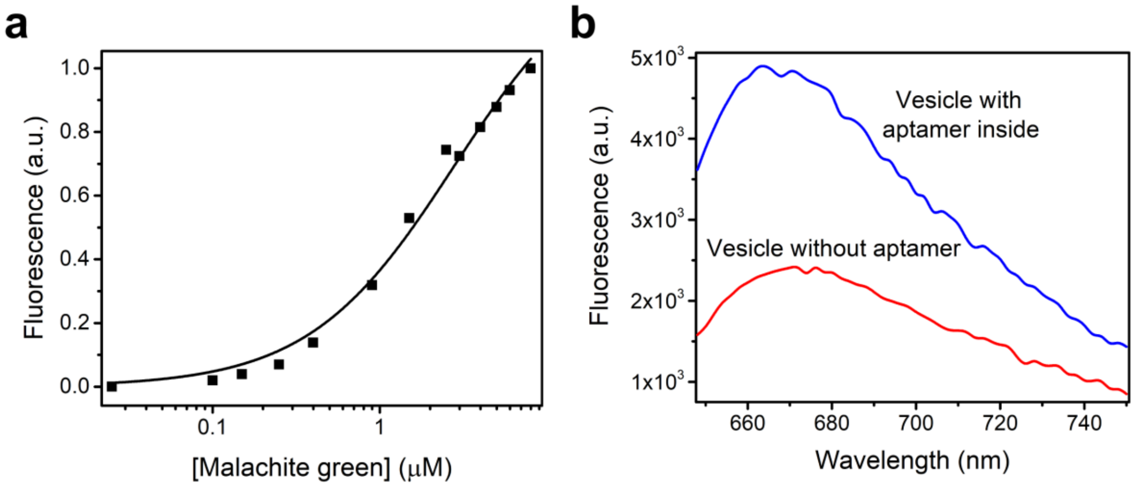

Supplementary Figure 2. Binding of the MG aptamer to MG. (**a**) Binding curve of MG aptamer to MG in the aqueous reaction buffer (10 mM HEPES, 1 mM Mg-Citrate, 100 mM KCl, 0.2 M bicine, pH 8.5) without vesicles. Solid line shows the curve fit to the Hill equation (see Methods) (K_D = 2.5 ± 0.4 μM). (**b**) Fluorescence emission spectrum of MG equilibrated with MA vesicles encapsulating aptamer (blue) or without aptamer (red). Raw fluorescence values are shown.

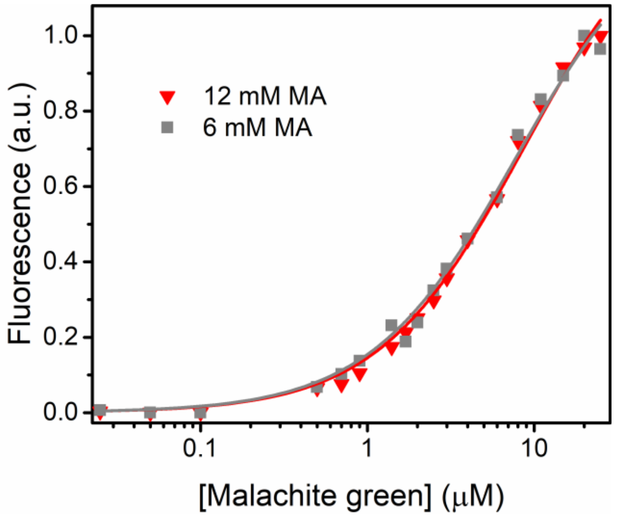

Supplementary Figure 3. Binding curve of MG aptamer in presence of different concentrations of MA. Solid lines shows the curve fit to the Hill equation (gray square: 6 mM MA, K_D = 6.8 ± 1.4 μM; red triangle: 12 mM MA, K_D = 8.6 ± 0.4). Values given are mean ± standard deviation.

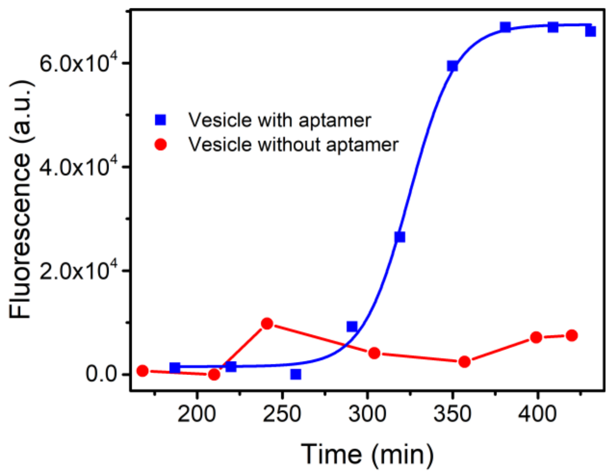

Supplementary Figure 4. Equilibration of MG across MA vesicle membranes. Equilibration was monitored by fluorescence, initiated by adding 15 μM MG to ~6 mM MA vesicles encapsulating the MG aptamer (blue) or without MG aptamer (red). For each run, the minimum fluorescence value ($\lambda = 660$ nm) was subtracted from all fluorescence values.

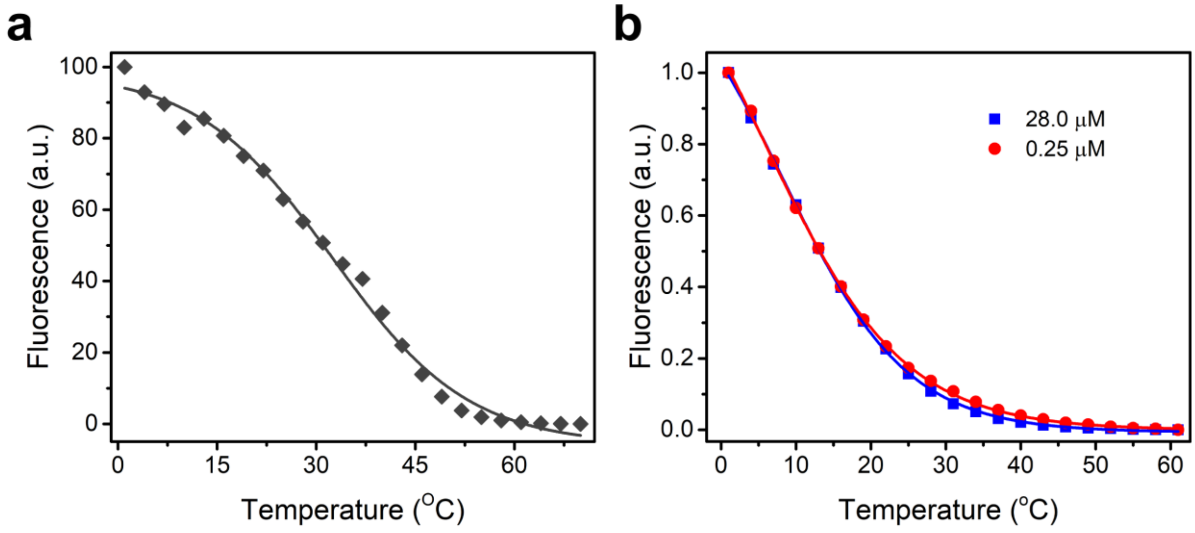

Supplementary Figure 5. Melting transition of the MG aptamer. Fluorescence response of the MG aptamer to temperature change in the presence of high MG concentration (~3-fold greater than the K_D) in (**a**) buffer without vesicles (10 mM HEPES, 1 mM Mg-Citrate, 100 mM KCl, 0.2 M bicine, pH 8.5) (T_t = 34.2 $\pm$ 2.3 °C), or **(b)** outside empty MA vesicles at different aptamer concentrations (indicated in legend).

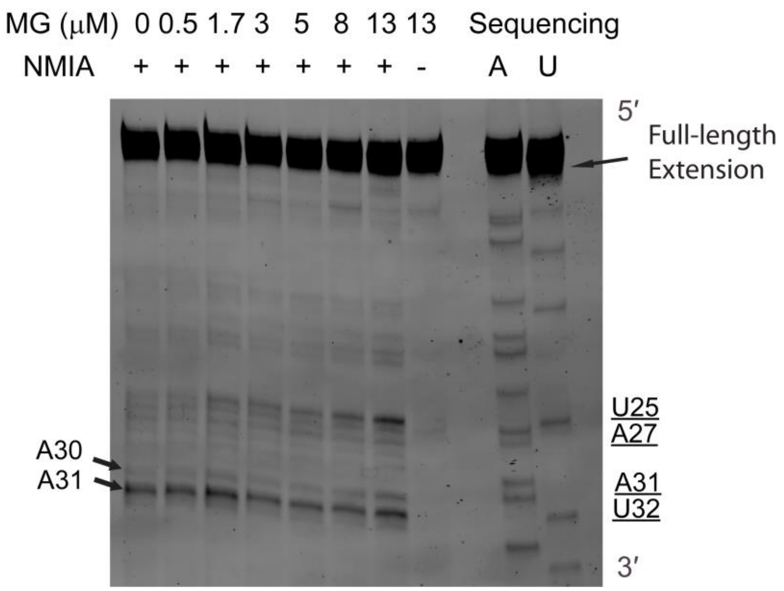

Supplementary Figure 6. Representative SHAPE gel of MG aptamer encapsulated inside MA vesicles. The first seven lanes show SHAPE reactions containing NMIA at increasing MG concentrations. The (-) lane shows a negative control containing 13 μM MG but in which no NMIA was added. The two rightmost lanes show A and U sequencing reactions performed using ddTTP and ddATP as chain terminators, respectively. The sequencing lanes give bands that are one nucleotide longer than the corresponding NMIA lanes.

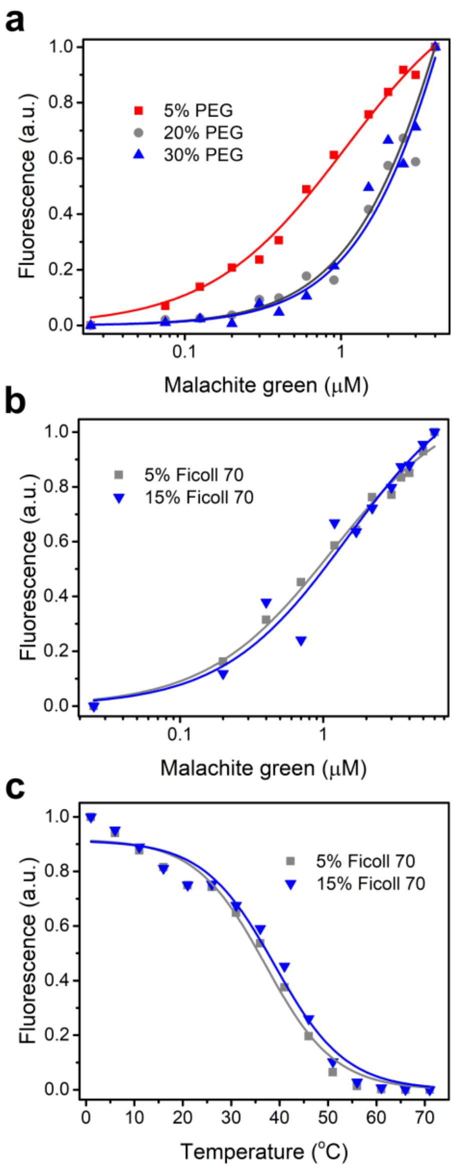

Supplementary Figure 7. MG aptamer in different concentrations of crowder molecules. Binding curve of MG aptamer in **(a)** PEG 8000 (in 5% PEG, K_D ~ 1.1 μM) and (**b)** Ficoll 70 (in 5% Ficoll 70, K_D ~ 1.5 μM; in 15 % Ficoll 70, K_D ~ 1.6 μM). **(c)** Melting transitions (T_t) of the MG aptamer (SHAPE construct) is also unaffected by Ficoll concentrations (5% and 15%, w/v).

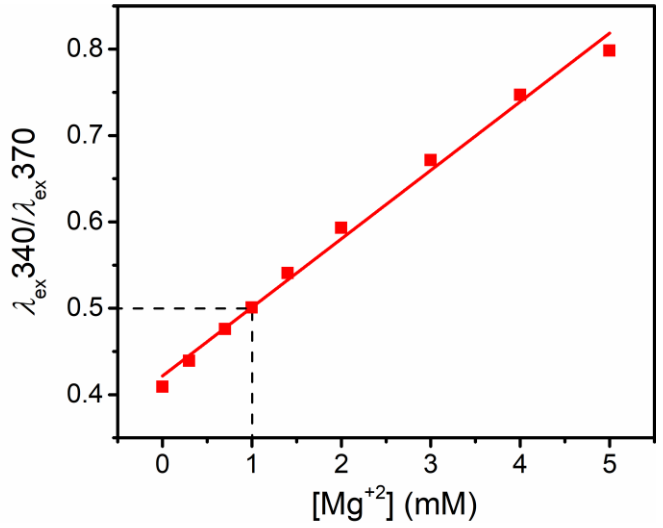

Supplementary Figure 8. Mg^{+2} concentration inside vesicle. Red: Standard curve for mag-fura-2 fluorescence excitation response to $[Mg^{2+}]$ in 10 mM HEPES, 1 mM $MgCl_2$, 100 mM KCl, 0.2 M bicine, pH 8.5. Dotted lines indicate the observed fluorescence of MA vesicles encapsulating mag-fura-2 and inferred $[Mg^{2+}]$.

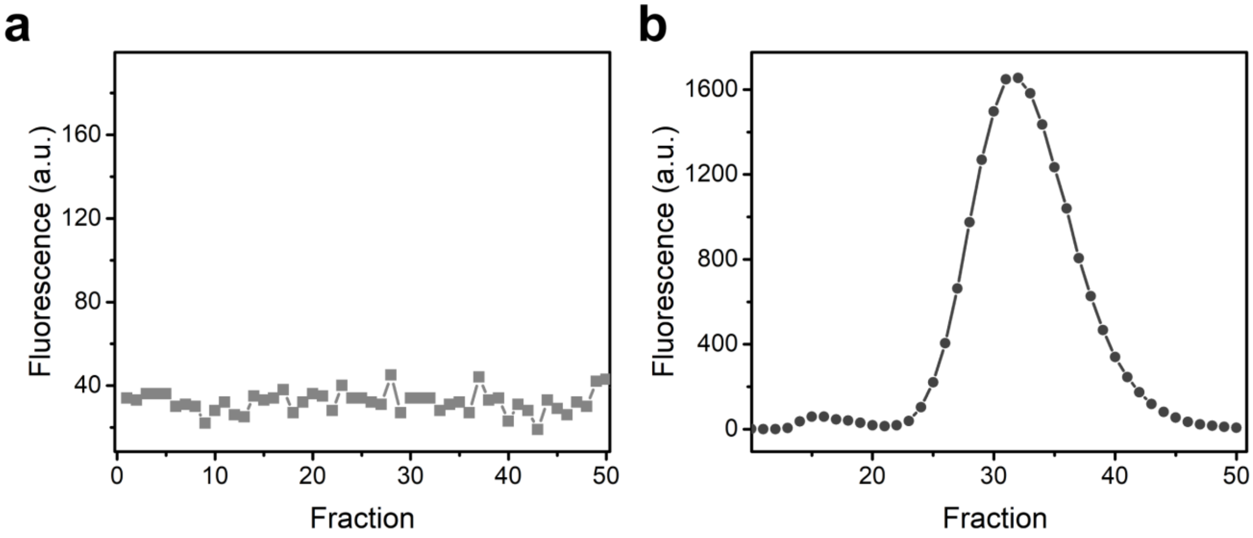

Supplementary Figure 9. Size exclusion chromatography of POPC vesicles with MG dye. 50 μM MG was added to 20 mM empty POPC vesicles and analyzed by SEC (**a**), or POPC vesicles formed in solution with the MG aptamer and purified by SEC (**b**). In (**b**), the peak corresponding to vesicles appears around fraction 15 and the peak corresponding to free aptamer appears around fraction 32.

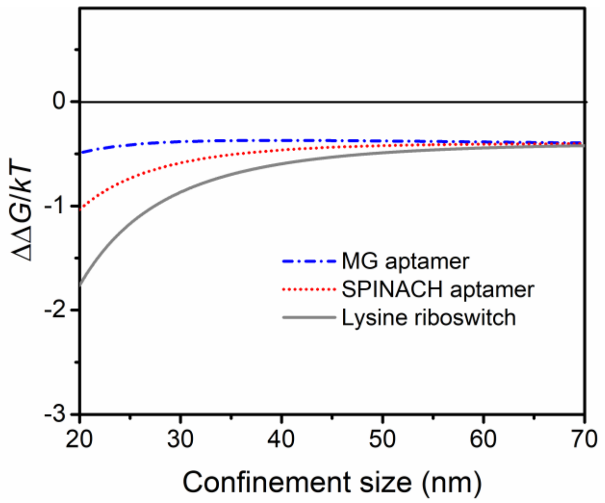

Supplementary Figure 10. Theoretical stabilization of RNA in confined spaces. Predicted effect of confinement on the free energy difference between the folded and unfolded forms, as a function of the vesicle inner diameter, following reference[1]. Three functional RNAs with known structures were used to estimate parameters for RNAs of different sizes. Parameters for the calculation: Kuhn length = 1.4 nm[2]; contour length = 0.63 nm per base[3]; MG aptamer (38 nt), $a_N \approx 1.7$ nm, $N = 17.1$ nm (PDB: 1Q8N[4]); SPINACH (91 nt), $a_N \approx 2.3$ nm, $N = 41$ nm (PDB: 4TS2[5]); Lysine riboswitch (161 nt), $a_N \approx 3$ nm, $N = 72.5$ nm (PDB: 4ERJ[6]), where a_N is the approximate radius of a sphere of equivalent volume and N is the contour length of the RNA.

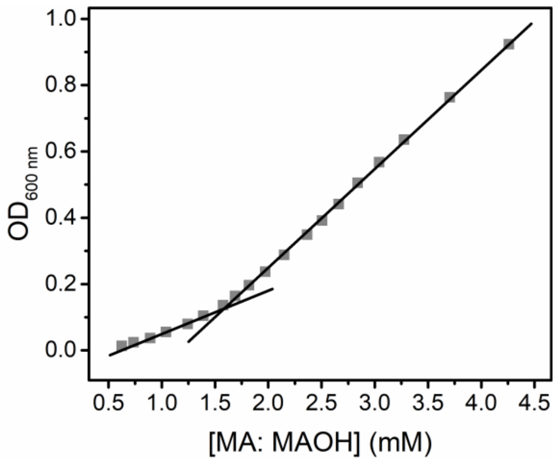

Supplementary Figure 11. Determination of CAC of MA:MAOH vesicle. The gradual dilution of non-extruded MA:MAOH vesicle suspension with aqueous buffer (10 mM HEPES, 1 mM Mg-Citrate, 100 mM KCl, 0.2 M bicine, pH 8.5) lowers the $OD_{600\ nm}$ of the solution. The point of inflection (~1.6 mM) was used to estimate the CAC of MA:MAOH.

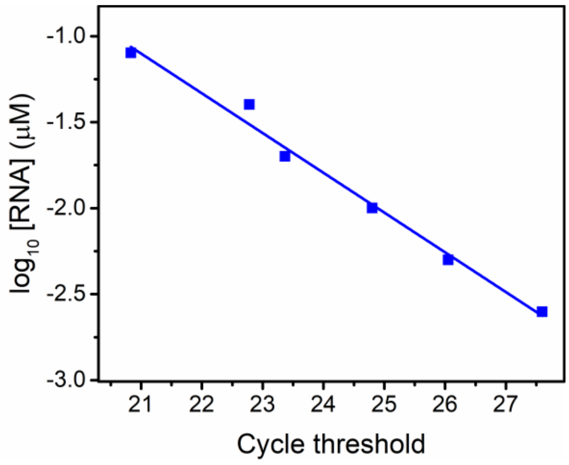

Supplementary Figure 12. RT-qPCR calibration curve. The RNA stocks were serially diluted to create the standard curve ranging from 0.08 μM - 0.002 μM. (solid line: linear regression line, $y = -0.23x + 3.8$, $R^2 = 0.985$).

Supplementary Table 1. Comparison of K_D values estimated by fitting to the Hill equation vs. exact quadratic solution.

**Sample**	**K_D (μM) (Hill equation)**	**K_D (μM) (Quadratic equation)**
In Aqueous Buffer	2.5 ± 0.4	2.4 ± 0.3
Outside MA Vesicles	6.8 ± 1.4	6.7 ± 1.3
Inside MA Vesicles	2.5 ± 0.2	2.6 ± 0.1
Outside MA:MAOH Vesicles	5.2 ± 0.1	5.3 ± 0.1
Inside MA:MAOH Vesicles	2.1 ± 0.2	2.0 ± 0.3
Outside MA:GMM Vesicles	5.1 ± 0.1	5.0 ± 0.1
Inside MA:GMM Vesicles	2.4 ± 0.1	2.3 ± 0.1
Outside DOPG Vesicles (with Mg^{+2})	20 ± 1.7	20 ± 1.6
Inside DOPG Vesicles (with Mg^{+2})	7.1 ± 0.2	7.3 ± 0.2
Outside DOPG Vesicles (without Mg^{+2})	20 ± 1	20 ± 0.4
Inside DOPG Vesicles (without Mg^{+2})	9.3 ± 2.4	9.5 ± 2.3
Outside POPC Vesicles	48 ± 8.6	48 ± 8.5
Inside POPC Vesicles	18 ± 1.4	18 ± 1.6
18% Glucose	3.6 ± 0.4	3.4 ± 0.4
18% Dextran	1.8 ± 0.4	1.7 ± 0.5

Supplementary References

1. Zhou, H.-X. & Dill, K.A. Stabilization of proteins in confined spaces. *Biochemistry* **40**, 11289-11293 (2001).
2. Camunas-Soler, J., Ribezzi-Crivellari, M. & Ritort, F. Elastic properties of nucleic acids by single-molecule force spectroscopy. *Annu. Rev. Biophys.* **45**, 65-84 (2016).
3. Murphy, M.C., Rasnik, I., Cheng, W., Lohman, T.M. & Ha, T. Probing single-stranded DNA conformational flexibility using fluorescence spectroscopy. *Biophys. J.* **86**, 2530-2537 (2004).
4. Flinders, J., *et al.* Recognition of planar and nonplanar ligands in the malachite green–RNA aptamer complex. *ChemBioChem* **5**, 62-72 (2004).
5. Warner, K.D., *et al.* Structural basis for activity of highly efficient RNA mimics of green fluorescent protein. *Nat. Struct. Mol. Biol.* **21**, 658 (2014).
6. Garst, A.D., Porter, E.B. & Batey, R.T. Insights into the regulatory landscape of the lysine riboswitch. *J. Mol. Biol.* **423**, 17-33 (2012).
